# Helping the helpers: mental wellbeing of the funeral professional

**DOI:** 10.3389/fpubh.2025.1721802

**Published:** 2025-12-11

**Authors:** Patricia Monroe, Didi Wu, Rachel Glassford, Lindsay Fuss, Jacey Kant, Sara A. Kohlbeck

**Affiliations:** 1 Medical College of Wisconsin Comprehensive Injury Center, Milwaukee, WI, United States; 2 Medical College of Wisconsin Department of Psychiatry and Behavioral Medicine, Milwaukee, WI, United States

**Keywords:** suicide, mental health, funeral professionals, vicarious trauma, suicide risk

## Abstract

**Introduction:**

This study aimed to understand the mental health challenges experienced by funeral professionals, in particular exposure to suicide. Additionally, we sought to understand what training related to suicide, suicide loss, and supporting individuals is acceptable to those in the profession.

**Methods:**

We developed a semi-structured interview guide with open-ended questions allowing for probing and additional follow-up inquiry. The main questions focused on three topic areas: (1) professional experience, (2) risk and protective factors of profession, and (3) exposure to suicide.

**Results:**

Through semi-structured interviews, four themes emerged: help-seeking, exposure to traumatic losses, value and limitations of peer support, and recommendations related to policy, culture, and training.

**Discussion:**

The study highlights the unique experiences and needs of funeral professionals that are often overlooked within discussions of vicarious trauma for first responder professions.

## Introduction

Occupational exposure to traumatic events can have a lasting impact on an individual's mental health and ability to function at work (1–3). Professionals exposed to suicide death have shown similar outcomes, with higher levels of exposure to suicides resulting in higher levels of depression, anxiety, post-traumatic stress disorder (PTSD), and suicidal ideation (4, 5). Much of this research focuses primarily on first responders (e.g., police officers, firefighters, emergency medical professionals) or mental health professions. Though exposed to similarly traumatic deaths in a stressful profession, the impact of occupational exposures on funeral directors' mental health is relatively unknown, especially in the United States. According to a 2018 survey, over 60% of funeral directors in the United States reported poor mental health in the previous 30 days due to stress, depression, or problems with emotions (6). Additionally, 36% of surveyed mortuary workers reported exposure to rude behavior daily (7).

High job demands, low job resources, secondary trauma and occupational stigma may be unique contributors to this profession's mental health, burnout, and risk for suicide (8–10). The Job Demands-Resources Model of Burnout predicts employee exhaustion and disengagement in environments with unfavorable shift-work schedules, demanding clients, time pressure, and low supervisor support which can be common in funeral homes (8). Outside of the funeral home, workers feel left out of family activities, 44.5% admitting discomfort attending events due to social stigma around the profession (7, 11). One study based out of Britain showed that, over the time period of 2001–2005, undertakers and mortuary assistants ranked #25 on the list of occupations with the highest suicide rates (12). Similar studies in the United States tend to not include funeral professionals as its own occupation, either conflating it with another occupational field or omitting it altogether (13, 14). With limited research, it is unknown if funeral directors are at a higher risk for suicide as compared with individuals in other occupations. In addition to occupational stigma, funeral directing is culturally known as men's work in the United States, particularly removals and embalming (11, 15). Research shows that women will likely have a harder time securing employment, emphasizing the importance of an experienced mentor in addition to existing training (11).

One mixed methods approach study conducted in Australia focused on postvention in the workplace, specifically examining a funeral company, gathering qualitative data concerning the workers' job responsibilities, opinions, and emotional impact of their career (16). This study revealed that many participants shared in the sorrow and grief of the family grieving a loved one who died by suicide and thus influencing their long-term wellbeing. Further, funeral professionals often interact with individuals soon after loss, including suicide, and those interactions can influence how individuals process the loss (17).

The present study sought to fill the gap in the literature on suicide risk of funeral professionals in the United States. The primary objective of this qualitative study is to assess the mental health and wellbeing of funeral home professionals in Wisconsin. It is well-known that healthcare and first response are occupational fields that exhibit higher suicide rates and risks. Knowing that greater exposure to traumatic deaths can increase mental health issues, including suicidal ideation, it is important to include funeral professionals in research. The second objective is to determine what training and supports funeral home professionals need to support those exposed to suicide loss. Findings from this study will inform suicide prevention measures for funeral professionals which could prevent suicides. Our research questions are as follows:

1. What work-related mental health challenges are experienced by funeral home professionals?
2. How has exposure to suicide impacted funeral professionals?
3. What type of suicide prevention training is needed by funeral home professionals to best support clients who have experienced suicide loss?

The objectives of this qualitative study are to (1) assess the mental health of funeral home professionals in Wisconsin, (2) determine what training and supports funeral home professionals need to support those exposed to suicide loss.

## Materials and methods

### Participants and procedures

Eligible participants were adults aged 18 or older, English-speaking, a Wisconsin resident, and work in the funeral home industry. A convenience sample of participants was recruited via social media and email outreach to funeral homes. Snowball sampling was also attempted with a focus on recruiting funeral professionals of different levels of experience and roles. In total, we interviewed 14 participants. Ten identified as women and four as men. Years of experience working in the field ranged from 1 to 40 (median = 15, mean = 12). The majority (*n* = 12) were funeral directors or apprentices, while two had purely administrative roles. Additional participant information is provided in Table 1.

**Table 1:** Participant information.

**Gender**	**Race/ethnicity**	**Years in profession**	**Role**
Woman	White	4	Director
Woman	White	1	Apprentice
Woman	White	3	Director
Woman	White	14	Director
Woman	White	18	Director
Woman	Hispanic	3	Apprentice
Man	White	17	Director
Man	Native American	40	Director
Woman	African American	7	Admin
Woman/non-binary	White	4	Admin
Man	White	15	Director
Man	White	15	Manager
Woman	Other-NHW/Asian	22	Director
Woman	White	1	Director

This qualitative project leveraged data from one-time, 1-h semi-structured interviews conducted in-person at an academic campus or online through Zoom. Interviews were conducted by one or two study personnel. Staff ranged from senior researchers to medical student research assistants. All staff were trained on the interview protocol.

An informational letter (in lieu of written consent) was shared with participants at the time the interview was scheduled. At the beginning of the interview, staff reviewed the informational letter in full again with participants. Once participants verbally expressed understanding and consent, audio recording through Zoom's recording feature or physical recorder was begun. After completion of the interview, participants received a $50 gift card. Audio recordings were subsequently transcribed using a word processing program, reviewed by staff for accuracy, and then deleted.

### Measures

We developed a semi-structured interview guide with open-ended questions allowing for probing and additional follow-up questions. The main questions focused on three topic areas: (1) professional experience, (2) risk and protective factors of profession, and (3) exposure to suicide. The interview guide was developed based on a strengths-based approach to validate lived experience of participants, explore the impact of their profession on their mental health, and determine exposure to suicide and suicide prevention trainings (see Table 2).

**Table 2:** Interview guide.

Task 1: professional experience • Please describe your work. • How long have you been working in this profession? • What do you find most rewarding about your work? Task 2: risk and protective factors of profession • Does your profession impact your mental health? If so, in what ways? • What are some common stressors that you or your colleagues experience in your profession? • Do the stressors that you encounter in your profession impact your relationships (e.g., family, friends)? If so, in what ways? • What are some ways you cope with stressors in your life? • What supportive resources are available through your workplace? Task 3: exposure to suicide • Have you ever experienced a personal loss from suicide? If so, how many? How long ago? How did this loss impact you? • Have you ever had to support a client(s) after a death from suicide? If so, how confident were you in your ability to support the individual(s)? What support did you provide to these clients? • Have you received any specialized training related to supporting others experiencing a mental health or suicide crisis? Task 4: wrap-up • Is there anything else you would like to share with me related to stress and mental health in your profession?

### Thematic analysis

Qualitative data was analyzed using an inductive thematic analysis approach. Thematic analysis is a flexible method of identifying patterns within data that builds on iterative levels of data organization (18). We utilized an inductive (“data-driven”) instead of deductive (“theory-driven”) approach as it's unknown if currently existing theories around workplace mental health apply to this professional field. In taking an inductive thematic analysis approach, we used the six-phase process as described by Braun and Clarke (19). These phases were followed through familiarization of data during transcription, generation of initial codes, searching for themes, reviewing themes, defining, and naming themes, and producing the write-up. Further interviews were not pursued once inductive thematic saturation as defined by Saunders et al. (20) was achieved.

The transcription and coding team consisted of four staff (PM, DW, RG, LF). PM developed a codebook containing definitions of each code which the coding team referenced for consistency. Each transcript was coded at least twice by separate team members. The coding team met before and after each round of coding to review the codebook and make updates as appropriate. Theme identification, reviewing, and defining were then conducted by three staff (PM, DW, LF). PM has a background in qualitative research, data collection and analysis. DW is a medical student who found easy parallels and distinctions between the professions of medicine and funeral service. LF is a public health professional with expertise in community-based interventions. PM, DW, and LF all identify as women with varying time and experience in higher academic institutions and research focused on mental health and suicide. These experiences may have influenced the research process, including interviews, analysis, and interpretation. To address this, consistent discussion and reflections on positionality were conducted throughout the analysis process.

### Ethics approval

The Institutional Review Board (IRB) at the Medical College of Wisconsin reviewed and approved all study activities. The IRB approved an informed consent process with the use of an informational letter for participants (PRO ID: 00042587). Study staff informed participants that all study activities were completely voluntary, and staff managed any participant distress by terminating interview, a debrief, and/or a warm hand-off to support services. Additionally, all participants received a handout of local mental health resources.

## Results

Our analysis identified four main themes. Identified theme, definition, and examples can be found in Table 3, with further information below.

**Table 3:** Interview themes, definitions, and participant quote.

**Theme**	**Theme definition**	**Sample quote**
Deeply entrenched occupational culture limits help-seeking and decreases wellbeing of funeral professionals.	Policies and expectations of the funeral professional, both internally and externally, limit the ability to seek help.	“The schedule is horrible. The circumstances are horrible. You know you're dealing with death every day. You have like families demanding things from you. You're stressed out because you're worried you forgot to do something. Or like you know, you don't see your family all the time. You're working on little to no sleep.”
Funeral professionals are expected to support others through grief yet sometimes feel ill-prepared to process sustained exposure to death, especially traumatic and personal losses.	Helping others is the primary focus for funeral professionals without training or understanding the impact on their own mental health.	“It was just, you know, a different situation where, you know, he was…they were your neighbors, you know. They offered you peaches off their tree, you know, that sort of thing.”
Peer support is valuable, though comes at a cost.	Peer support is readily available and valued but may not effectively address mental health challenges.	“Probably definitely feel about my coworkers, I feel like they are my loved ones.”
From the individual to the macro, a shift in systems may increase the wellbeing of funeral professionals.	Increased benefits, targeted training, and a robust peer support program is recommended to increase mental wellbeing.	“Maybe just an incentive. Maybe you get paid more if you're on call or if you have to work late. Or even just having another day off. I think that would be the best for supporting the employees.”

### Theme 1: deeply entrenched occupational culture limits help-seeking and decreases wellbeing of funeral professionals

Policies relating to work schedules, in particular the on-call schedule, and pay and benefits were almost unanimously described by all interviewees as impacting funeral professionals' health and wellbeing. The influence of this logistical aspect of their role is compounded by the physical and emotional responsibilities of the job. While individuals often viewed their role as a “calling” and find the rewards of supporting families as motivating, the time-sensitive demands and the needs of grieving families take a toll.

“The schedule is horrible. The circumstances are horrible. You know you're dealing with death every day. You have like families demanding things from you. You're stressed out because you're worried you forgot to do something. Or like you know, you don't see your family all the time. You're working on little to no sleep.”

The strong desire to help families can drive a sense of perfectionism and need to dissociate their own feelings and needs.

“And just like the emotional load of, like, you're literally with grieving people all day and all night. If you're an empathetic person, that would be very draining. And you're like, oh my gosh, my life is literally full of depression because these people are all so sad. Like, you're carrying that energy with you through every single day. So not having the time to release that energy is incredibly difficult.”

Further, culture within and outside of the profession, including expectations around the support funeral professionals provide to the bereaved, can inhibit help-seeking and lead to isolation. This is highlighted by several funeral professionals.

“To like pick up the slack a little bit because I honestly cannot do this today. I need to take a day off. I need a mental health day. And that's just not something that is acceptable in the profession at this point.”

“Funeral directors are again wired differently. We're not supposed to ask for help. People come to us for help.”

“We're sort of like stuck in the middle. Nobody really knows where to categorize us or where to put us or what. It's kind of a weird profession. That I think doesn't get taken seriously or get paid a lot of attention.”

### Theme 2: funeral professionals are expected to support others through grief yet sometimes feel ill-prepared to process sustained exposure to death, especially traumatic and personal losses

When speaking about the types of cases that they encountered, funeral professionals shared that several traumatic types of deaths can particularly affect their own mental health and wellbeing. Suicides and deaths of youth were identified most often.

“But that's the toughest part…. And I think we're always inclined, as you know, to have something to offer, but really in those cases, you know the death of a child. I mean…I'd have to tell people. You know, there's really nothing. Nothing I can say to you right now.”

Suicide exposure, either through the profession or personal, was noted by all interviewees, with three individuals describing the loss of a coworker by suicide.

“I know it's difficult. There was someone, not in our bubble, but that my coworkers know that did commit suicide in the past year, which was really hard for my coworkers. To some of them, went to school with them. And they really struggled with that loss, even though they work in this industry. So that was difficult.”

Even if the loss was not due to suicide, the type of loss or the individual who died, may impact funeral professionals' mental wellbeing. This can be especially true for funeral professionals who work in small communities. One individual described an experience of needing to care for someone they knew:

“It was just, you know, a different situation where, you know, he was…they were your neighbors, you know. They offered you peaches off their tree, you know, that sort of thing.”

Though it is expected to deal with death on a daily basis, funeral professionals explained that their training did not prepare them adequately with self-efficacy often coming from on-the-job experiences. This was highlighted by one funeral professional:

“When I was in school, as a new person entering this industry, it wasn't really taught to me…one day, you know, they're like, hey, there is a 9:00 appointment for a family who the husband committed suicide. Go for it. And you're like, OK, you know, so you just kind of get thrown in.”

### Theme 3: peer support is valuable, though it comes at a cost

Funeral professionals value the connections and relationships they develop within the workplace, acknowledging that it is often professional peers that truly understand the aspects of their role and impact of daily responsibilities on physical and mental wellbeing. Often, these relationships offer a source of comfort, understanding, and companionship. One funeral professional noted:

“Probably definitely feel about my coworkers, I feel like they are my loved ones.”

Even though peers are consistently a source of support, the persistent reliance on each other for emotional release may compound stress. This is highlighted by one funeral professional stating:

“I've got a couple of friends that are, you know, funeral directors obviously too. And you know we always speak to each other. But at the same time too. I don't know how healthy that is as well, because, you know, we're all stressed out and you know, we all need our breaks too.”

Additionally, natural coping skills that surface within the profession may perpetuate negative health outcomes. Several funeral professionals noted substance use, in particular alcohol, as a common coping mechanism that has been engrained in the culture of the profession.

“A lot of my colleagues, myself included, we drink way too much. It seems like alcohol is pretty common for most of us. Just because we need to shut our brains off before we can go to bed or whatever. My day off I‘ll still be working. A lot of us are, just because it's… So in order to just stop and stop thinking about work, a lot of us use alcohol.”

Another funeral professional noted the persistent negative tone and disassociation shared amongst coworkers.

“Keep pushing it down and don't deal with your own emotions about all this. Keep going. You can, you know, or we would joke that we didn't have a soul left anymore because we were just so downtrodden. And beaten down by being the funeral director that you know, we were left with nothing of our own…we would have a lot of dark humor, to kind of cope with stressful situations and times, I guess.”

### Theme 4: from the individual to the macro, a shift in systems may increase the wellbeing of funeral professionals

A majority of funeral professionals provided recommendations related to policies and the culture of their profession that could improve the wellbeing of their colleagues and themselves. Of note, schedule adjustments, especially for apprentices, was stated often, along with improved pay and benefits.

“Maybe just an incentive. Maybe you get paid more if you're on call or if you have to work late. Or even just having another day off. I think that would be the best for supporting the employees.”

For individuals newer to the profession, a need for a shift in culture related to mental wellbeing was noted. Recognition of mental health care and a work/life balance was identified as important to promote across all levels of funeral professionals.

“I think like providing like maybe like a free therapy session every other week. Or like something of that nature. Or wellness check-ins. Like what can we do to support you? Or like staff meetings like, what do the apprentices need? How are you guys feeling like?” Has anything been affecting you lately? So, what can we do to help you? You know.

Funeral professionals endorsed a need for access to training on suicide, suicide loss, and how to support individuals who are bereaved. However, training should fit the needs of learners, including experiential learning to increase self-efficacy and engagement.

“Anything else that you know would better our job to serve families is great. Versus having to say, you know, not really like this… But like, sorry for your loss. Here's some, you know, brochures like best of luck to you. If there's just certain things where you know how to suggest how to handle.”

Finally, funeral professionals reiterated the importance of having someone to speak to that truly understands the profession. This could be a clinician with a background or connection to the profession, or implementation of structured peer support.

“…they would have somewhere to go, that they could actually talk to somebody independently and say, yeah, I know. Exactly what you're I know what you're going through. I've gone through it or I've seen, or I've helped other people get through this. I think that would be, I think I'd be invaluable and honestly, I really do.”

“In recognizing that the hardest thing about You know anybody that deals with grief at such a high capacity, whether that's er or funeral directors? Is that just turning to each other and asking, Are you okay?”

## Discussion

This study aimed to understand the mental health and wellbeing of funeral professionals. Also, we examined challenges experienced by funeral professionals as they relate to exposure to traumatic losses like suicide. Finally, we sought to understand what, if any, training related to suicide, suicide loss, and supporting individuals is needed and/or acceptable to individuals in the profession. Our findings point to a social ecological view of stressors and support that impact and influence the mental health and wellbeing of funeral professionals, as factors often outside the individual, including cultural factors and policy related factors, play a significant role. This highlights the need for structural and cultural changes within the funeral home profession to better support the workforce (21). Additionally, this study highlights the unique experiences and needs of funeral professionals that are often overlooked within discussions of vicarious trauma for first responder professions, and reflects the components of the Job Demands-Resources Model leading to exhaustion and disengagement (8).

### Help-seeking and wellbeing of funeral professionals

A majority of participants noted the stress of the on-call schedule impacting their ability to participate in activities that are protective for individual mental health. This can enable a culture that overextends workers and inhibits help-seeking (22), either by not having the time to seek support or pressuring funeral professionals to “push through.” Further, a misperception of this role and daily responsibilities from individuals outside the profession may lead funeral professionals to delay or avoid help-seeking. In some ways, the stress and workload of the funeral profession is similar to what is experienced in other medical professions, and particularly for trainees, including medical residents (23). These beliefs, norms, and values lead to an occupational culture that goes beyond a single workplace, extending to anyone in the profession and perpetuating negative health behaviors and outcomes (24, 25).

### Grief support and exposure to traumatic losses

Vicarious trauma occurs when individuals are exposed repeatedly to trauma second-hand (e.g., through listening, reading, viewing), often due to profession, that results in negative mental and physical health outcomes. Due to their increased exposure, occupations like first responders, victim services, medical specialists, and other allied professions are considered at risk for experiencing negative reactions (26). Funeral professionals naturally deal with death and loss routinely in their roles, with increased exposure to traumatic deaths (27). This experience was highlighted by participants in this study. This possibly signals higher rates of suicide that is not reflected in current data on suicide risk for funeral professionals (12). Additionally, funeral professionals are expected to provide grief support to families, often with limited training, as noted by participants and reflected in the literature (17). This is also seen in the medical profession, an occupation that has similar exposure to death and dying (28). Depending on location, funeral professionals may also know the decedent or decedent's family. Familiarity and the number of exposures to death create a unique grief experience for the funeral professional themselves (29).

### Accessibility and limitations of peer support

Social connection has been found to be a protective factor for negative mental health outcomes and suicide (30). Participants in this study noted peers as a powerful support, especially having someone that understands their professional duties and related stress. However, participants also noted that relying on each other could create additional burden, as well as reinforce negative coping strategies. The benefit of peer support can also be limited dependent on the culture of the funeral home. A sense of belonging and community within a workplace has demonstrated higher levels of quality of life and resiliency (31). Participants, both new to the field and seasoned veterans, noted generational differences in mental health and approaches to dealing with work stress. Further, some participants noted the shift from family-owned funeral homes to corporate owned, leading to a disconnect between leadership and individuals on the ground.

Findings from this study also align with the three elements of the Interpersonal-Psychological Theory of Suicide (IPTS) (32). Thwarted belongingness is experienced in our sample through the long work hours and high demands of these professionals, creating distance between funeral professionals and the much-needed support of family and other close loved ones. Perceived burdensomeness is also observed through the sense that while peer support may be helpful in addressing the various challenges experienced by funeral professionals, these individuals are reticent to reach out to others out of fear of burdening them. This leads to the internalization of the painful realities of this profession, which may exacerbate stress or other mental health challenges. Finally, funeral professionals are exposed to painful and provocative events as part of their work, including providing care for deceased children. The IPTS posits that repeated exposure to such events can lead to the development of capability for suicide (33), another component of the model. The alignment of the experiences of funeral professionals with the IPTS highlights the potential risk for suicide among this group as well as the need for practical, relevant prevention strategies within this profession.

### Implications for practice and future research

Several suggestions for improving the mental health of funeral professionals were identified in our study. While access to resources is important, policy, culture, and training may make a bigger impact. First, organizational policies limiting work hours for apprentices, similar to medical trainees, along with increased pay and benefits, could be beneficial. Promotion and even modeling of help-seeking from organizational leadership may also increase positive coping strategies among funeral home personnel while reducing stigma and normalizing mental health challenges. Organizational leadership could consider integrating discussions around mental health into pre-existing meetings or supporting wellbeing initiatives at work. Organizations may also consider the inclusion and codification of “mental health days” as part of employment benefits and personal time off. Another example could include providing time off during difficult anniversaries—for example, an anniversary of an impactful death or suicide—to make space for processing and support. Training for funeral professionals related to grief and supporting others, as well as community education on the role of funeral professionals and the impact of their role, could promote a sense of self-efficacy and understanding among community members. Creating programing that allows structured peer support and open dialogue between funeral professionals, and their support systems could reduce the isolation and stigma experienced. This could be accomplished through training interested peers in Emotional CPR (eCPR) or Mental Health First Aid and then implementing regular peer check-ins between trained professionals and their colleagues. Finally, improved data collection related to occupation within the violent death reporting system and other surveillance systems will provide a more accurate reflection of the burden of suicide within the profession. This could be accomplished by standardizing occupation data that is reported on death certificates and/or allowing for more than one occupation to be reported on death certificates. Further research on mental health outcomes, including suicidal thinking and behavior, of funeral professionals is needed.

### Limitations

While novel, this study has limitations to consider. This study reflects the experiences of Wisconsin funeral professionals, and therefore the findings may not be generalizable to all funeral professionals. We attempted to recruit study participants from across the state, with representation from both urban and rural settings, and study participants reflected multiple racial and ethnic backgrounds, LGBTQ status, and years within the profession. There is also a possibility of selection bias in this study, as individuals who are more interested in issues relating to mental health and wellness may have been more likely to respond to study recruitment. This is potentially reflected in the number of women who completed the interviews, despite the predominately male occupation.

## Conclusion

This study provides insight to the impact of the role of funeral professionals on their mental health, a profession often overlooked when discussing vicarious trauma, compassion fatigue, and suicide risk. Similar to other first responders and medical trainees, funeral professionals experience long work schedules, exposure to traumatic experiences, limited pay and benefits, and inconsistent support. They are also driven by caring for others, value support from peers, and should be considered when developing or advocating for mental health funding and support.

## Data Availability

The raw data supporting the conclusions of this article will be made available by the authors, without undue reservation.
